# Early-Age Running Enhances Activity of Adult-Born Dentate Granule Neurons Following Learning in Rats

**DOI:** 10.1523/ENEURO.0237-17.2017

**Published:** 2017-08-16

**Authors:** Olga Shevtsova, Yao-Fang Tan, Christina M. Merkley, Gordon Winocur, J. Martin Wojtowicz

**Affiliations:** 1 Department of Physiology, University of Toronto, Toronto, Ontario M5S1A8, Canada; 2 Rotman Research Institute, Baycrest Centre, Toronto, Ontario M6E2E1, Canada; 3 Department of Psychology, Trent University, Peterborough, K9J7B8, Canada

**Keywords:** adult neurogenesis, dentate gyrus, hippocampus, learning and memory, plasticity

## Abstract

Cognitive reserve, the brain’s capacity to draw on enriching experiences during youth, is believed to protect against memory loss associated with a decline in hippocampal function, as seen in normal aging and neurodegenerative disease. Adult neurogenesis has been suggested as a specific mechanism involved in cognitive (or neurogenic) reserve. The first objective of this study was to compare learning–related neuronal activity in adult-born versus developmentally born hippocampal neurons in juvenile male rats that had engaged in extensive running activity during early development or reared in a standard laboratory environment. The second objective was to investigate the long-term effect of exercise in rats on learning and memory of a contextual fear (CF) response later in adulthood. These aims address the important question as to whether exercise in early life is sufficient to build a reserve that protects against the process of cognitive aging. The results reveal a long-term effect of early running on adult-born dentate granule neurons and a special role for adult-born neurons in contextual memory, in a manner that is consistent with the neurogenic reserve hypothesis.

## Significance Statement

The role of adult neurogenesis in learning and memory is under active investigation, but the underlying mechanisms remain unclear. The present study found that early-age running led to enhanced associative learning and memory in adult rats and increased activity of adult-born granule neurons in the dentate gyrus (DG) during memory retrieval. This study demonstrates the long-term effect of early-age physical activity on learning and memory much later in life. The findings emphasize the involvement of adult-born hippocampal neurons in neurogenic and functional cognitive reserve and show that physical activity contributes to memory improvement.

## Introduction

Cognitive reserve refers to the brain’s capacity to draw on enriching experiences during youth to protect against adverse effects of structural decline, as in normal aging, and neuropathological damage resulting from accident or disease (Stern, 2002). A number of factors, including physical exercise, education (Puccioni and Vallesi, 2012), occupation, and lifestyle (Fratiglioni and Wang, 2007), contribute to cognitive reserve, which has been viewed as a compensatory mechanism for optimizing function in the compromised brain.

The relationship between cognitive protection and brain plasticity is central to the concept of cognitive reserve but our understanding of specific brain mechanisms involved is limited. One possibility may relate to adult neurogenesis, the capacity to produce new cells in the hippocampus that become integrated into neuronal networks of learning and memory. Several investigators have shown that physical exercise and other types of environmental enrichment increase neurogenesis levels (van Praag et al., 1999) and improve performance on hippocampus-sensitive tests of cognition (Farmer et al., 2004). Based on these findings, Kempermann proposed a “neurogenic reserve” hypothesis in which the potential for adult neurogenesis is maintained. According to this hypothesis, the continued exposure to stimulating events in young age leads to the increased production of new hippocampal neurons to support cognitive function in old age (Kempermann, 2008).

In previous studies, we used voluntary running to increase the levels of hippocampal neurogenesis in juvenile rats and examine neurogenesis levels at various time points, up to nine months later (Merkley et al., 2014). Voluntary running was chosen as an enriching activity because of its known effects on neurogenesis (van Praag et al., 1999), and associated effects on learning and memory (van Praag et al., 2005; Abrous and Wojtowicz, 2016). Our study provided the first evidence that early life physical activity in rodents can build a long-lasting neurogenic reserve later in life (Merkley et al., 2014).

The question addressed in the present study is whether voluntary running during early development can contribute to such a process in later adulthood. To test whether neurogenic reserve serves as a mechanism to improve memory, one group of rats was given voluntary access to running wheels for six weeks and another group was housed in standard laboratory cages. After four months rats were trained on a contextual fear (CF) conditioning task and, two weeks later, tested for memory of the CF response. CF is known to depend on the hippocampus and, in particular, new hippocampal neurons (Winocur et al., 2006; Winocur et al., 2012). Rats received CF conditioning when they were six to seven months old. At that age, a potential reserve mechanism would be primed for possible use in case of damage or disease, but not yet engaged in a compensatory manner.

Testing was done in the original conditioning context, a similar one, or a very different context, to provide information on the quality of memories. The purpose of manipulating context at test was to determine if these effects generalized to contexts that bore some similarity to the conditioning chamber but were not associated directly with shock. A novel aspect of the present study was the utilization of c-Fos-immunoreactivity to measure the activity of adult born neurons in the dentate gyrus (DG) in response to testing. Using immunohistochemical methods with a mitotic marker 5-chloro-2’-deoxyuridine (CldU) and an activity marker c-Fos, we were able to demonstrate the enhanced activity of adult-born dentate granule neurons in comparison to developmentally born neurons.

## Materials and Methods

### Experimental design

One-month-old (juvenile) male rats (*n* = 80) were housed for six weeks in specially designed cages with free access to running wheels, or in standard laboratory cages. After one week of acclimatization, the rats were divided into two groups, runners (*n* = 40) and non-runners (*n* = 40), and kept in single cages throughout the study. Runners and non-runners were maintained under the same housing conditions, including ad libitum access to food and water, except that cages of runners were fitted with a running wheel (circumference: 1.07 m). Running was monitored daily by means of a Vital View data acquisition system (Mini-Mitter A Respironics Company).

After six weeks, the running wheels were removed and the animals housed in pairs for four months in standard laboratory cages conditions for the remainder of the experiment (see below). The running and non-running groups were subdivided into four subgroups. (1) No training and no test (controls). (2) Context A. (3) Context A’. And (4) context B. Three weeks before CF conditioning, all rats received a CldU injection. When the rats were six to seven months old, they were trained on a standard form of CF conditioning (Winocur et al., 2006). Two weeks later, memory for the fear response was tested in the original conditioning chamber (context A), a similar chamber (context A’), or a very different chamber (context B).

**Figure 1.**
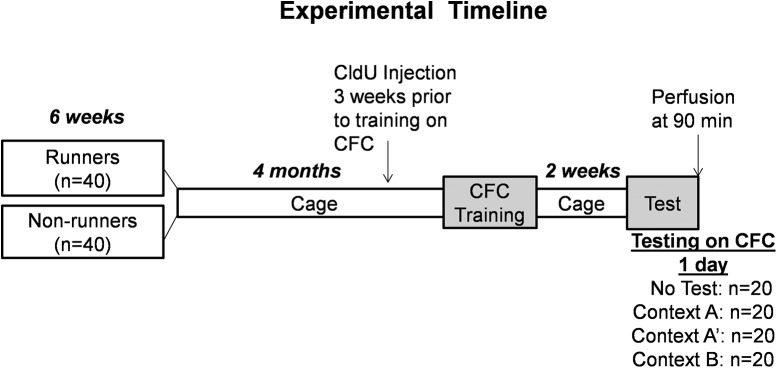
Experimental timeline. At one month of age, a group of 40 rats was exposed to running wheel while the other group was kept in standard cages for six weeks. All rats were trained on the contextual fear conditioning (CFC) task in context A. Two weeks after training, 10 rats from each group were tested in context A, A’, or B. The remaining rats served as untested controls. Mitotic marker CldU was injected three weeks before training. Ninety minutes after the test, all animals were perfused for immunohistochemistry.

Animals were killed shortly after testing, and adult neurogenesis in the DG of the hippocampus was analyzed. Training and testing environments, as well as tissue collection, processing, and immunohistochemistry, are described in detail below. The experimental timeline is shown in Figure 1.

### Animals

Long Evans rats (Charles River) were used in this study. All animals were maintained on a 12/12 h light/dark cycle with lights on at 7 A.M. to 7 P.M. Animal weights were recorded regularly and animal procedures were in accordance with the guidelines of the Canadian Council on Animal Care. The experimental protocol was approved by the Animal Care Committee at the University of Toronto and Trent University.

### Running

Running distances were monitored daily as well as the time of day that each animal spent running. The records showed that the rats predominantly ran during the dark phase of their cycle. Cumulative running distance or average distance traveled did not differ between cohorts designated to testing and control groups but the distance covered across the entire running period of six weeks increased progressively for all groups. Thus, rats ran an average of 21.8 (SD = 14.8) km during week 1 and reached the maximum of 71.1 (SD = 57.9) km during weeks five and six.

### Training and testing environments

All rats received CF conditioning in a wooden chamber (50 × 40 × 18 cm) that had four walls made of clear Plexiglas, a hinged clear Plexiglas roof with holes to allow ventilation, and a floor that consisted of metal rods, spaced 1.3 cm apart. The chamber was placed on a table, 1.3 m above the floor, and situated in the center of a standard laboratory room. The room contained standard furniture (e.g., desk, table, bookshelf along one wall, etc.), as well as pictures, light fixtures, etc. on the walls. Illumination was provided by overhead fluorescent lights under rheostatic control.

Training procedures for CF conditioning were similar to those followed in previous studies (Winocur et al., 2006; 2007). Each rat received one fear conditioning trial that began with the rat being placed in the chamber and allowed to explore freely for 5 min. Near the end of the exploration period and over a 64-s period, eight observations of freezing behavior were recorded every 8 s to obtain a preshock measure of freezing. Freezing was defined by an immobilized crouching response in which the only detectable movement was the rat’s breathing. Behavior was monitored by an overhead video camera connected to a recorder and data processing system that recorded the time spent freezing. The rat then received 10 tone-shock pairings at 2-min intervals (tone: 2000 Hz; 80–90 db, 30 s; shock: 1.5 mA; 1 s). The tone was presented through a centrally mounted speaker attached to the box, and the shock was delivered by TechServe (Model 452A shock generator). Beginning 30 s after the last shock and over a 64-s period, freezing behavior was recorded every 8 s (eight observations). One minute later, the rat was removed from the box and returned to its home cage.

For testing, rats assigned to context A were tested in the same chamber and environment as in CF conditioning. Rats in context A’ were tested in the same chamber but the environment was slightly changed (e.g., room objects rearranged, lighting dimmed slightly). Rats in the context B were tested in a smaller box (40 × 30 × 18 cm), also made of Plexiglas but with walls that were lined with opaque gray material. The roof was clear Plexiglas with ventilation holes and the floor consisted of metal rods, spaced 1.3 cm apart. This test box was placed in a different room on a table that was situated against a wall. Care was taken to ensure that the configuration of furniture, pictures, etc., was different from that of the room in which fear conditioning took place. Testing procedures were identical in all conditions. Testing consisted of a single trial in which the rat was placed in the appropriate box for 8 min, and in the absence of the tone, the amount of time spent freezing was recorded. The rat was then removed from the box and returned to its home cage.

#### CldU injections

Three weeks before contextual fear conditioning (CFC) training, each rat was injected intraperitoneally with thymidine analog CldU (105478, MP Biomedicals). The injected solution was prepared by dissolving CldU in saline at 10 mg/ml and adjusting the pH to 7 with 0.5 µl of 10 N NaOH/ml saline. The injected dose was 85 mg of CldU/kg.

### Tissue collection and processing

Ninety minutes after testing animals were deeply anesthetized with isoflurane inhalation followed by transcardial perfusion with 300 ml ice-cold PBS followed by 300 ml ice-cold 4% paraformaldehyde (PFA). Following decapitation, brains were removed and placed in PFA for 24 h at 4°C. After postfix, brains were placed in PBS containing 0.1% sodium azide and stored until further processing.

### Immunohistochemistry

Brains were cut in half, and the hippocampus was dissected from the right hemisphere in each animal. Isolated hippocampi were sectioned along the dorso-ventral axis using a vibratome (VT1000S, Leica Microsystems) into sections 30 μm thick. The sections were stored in PBS containing 0.1% sodium azide at 4°C until staining. Twelve sections were selected from each animal using a systematic random sampling procedure previously described (Wojtowicz and Kee, 2006). All immunohistochemistry was conducted on free floating sections. Importantly, sections were rinsed extensively in PBS before processing and between each incubation. All primary and secondary antibody incubations were conducted in a PBS solution containing 0.3% Triton X-100. In experiments involving labeling of CldU, sections were incubated at 45°C for 30 min in HCl (1.0 N) to denature DNA and unmask the antigen before incubation in primary antibody, preceded and followed by extensive rinsing.

### Detection of c-Fos and CldU

To identify cell survival and activity of newly formed cells, double-label immunohistochemistry was conducted for CldU and immediate early gene (IEG) protein c-Fos. Sections were incubated sequentially, with primary anti-c-Fos antibody (1:2000, 72 h at 4°C; Millipore, ABE457), followed by secondary antibody donkey anti-rabbit IgG Alexa Fluor 568 (1:200, 2 h at room temperature (RT), Life Technologies, A10042) in dark; afterward, to detect CldU, the tissue was incubated with primary antibody rat anti-BrdU (1:1500, 24 h at 4°C, AbD Serotec, OBT0030), followed by secondary antibody goat anti-rat IgG Alexa Fluor 488 (1:200, 2 h at RT, Life Technologies, A11006). This particular antibody can bind to CldU with high affinity (Merkley et al., 2014). In all experiments, sections were mounted onto glass slides using double-distilled water (ddH_2_O), and coverslipped using PermaFluor (Thermo Scientific). Immunohistochemical controls included the omission of primary antibodies, which resulted in lack of staining at the corresponding wavelength in each instance.

### Quantification of cells and cell counts

All the single immunolabeled cells in the granule cell layer (GCL) zone of the DG were counted using a fluorescent microscope (Nikon, Eclipse Ni). Double-labeled neurons were visualized using Leica TCS SP5 confocal microscope (Leica Microsystems). The average number of cells per sampling section was multiplied by the number of hippocampal sections to yield the total number of cells per DG in each rat. Twelve sections were initially sampled from each animal. The sampling was repeated twice or in some animals three times to reach a minimum of at least 30 CldU+ cells per animal. On average 68.3 (SD = 34.3, *n* = 80) CldU+ cells per animal were examined for double-labeling with c-Fos.

### Statistical analysis

For immunohistochemistry and behavioral data, differences between groups were analyzed using two-way ANOVA with running and testing as variables. All pairwise multiple comparisons were conducted using the Holm-Sidak *post hoc* test or, in case of behavioral data, the Bonferroni multiple comparisons procedure. All values were expressed as mean ± SEM or SD when appropriate. Statistical analyses were performed using Sigma Plot 12.0 software (Systat Software). The level of statistical significance was set at *p* ≤ 0.05.

## Results

### Hippocampal neurons are activated during a contextual learning task

**Figure 2.**
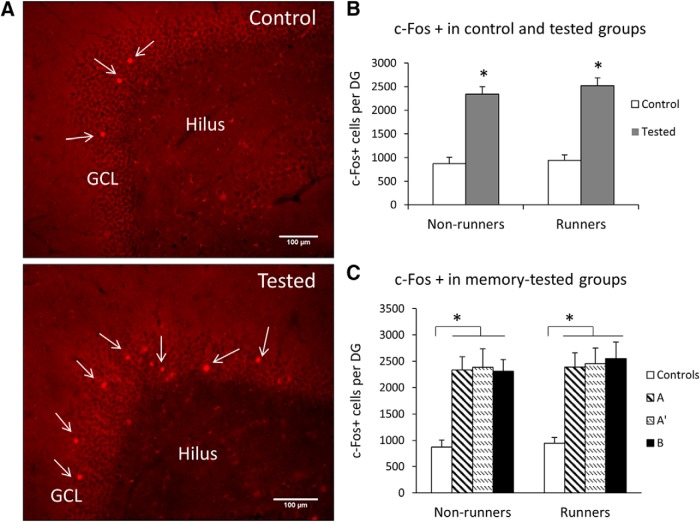
c- Fos activity in DG neurons. ***A***, Fluorescent microscopic images showing c-Fos cells in the DG of control and tested rats. White arrows indicate c-Fos-labeled cells in the GCL of the DG. ***B***, The number (mean ± SEM) of c-Fos-labeled cells per DG. Tested animals showed significantly more c-Fos+ cells per DG than controls (*p < 0.05). A similar result was obtained for the CA1 field of the hippocampus (Fig. 2-1, Extended data). ***C***, Running and individual tested groups. Controls, non-tested cage controls; A, tested in the familiar environment; A’, tested in the similar environment; B, tested in the novel environment. The number of c-Fos+ cells was greater in all tested groups compared to controls in runners and non-runners (*p < 0.05, *n* = 10 rats/group).

A necessary element of this study is a measure of overall activity in the hippocampal neurons, especially those in the DG. For this purpose, we used a c-Fos protein detected by immunohistochemistry as a measure of c-Fos gene expression. The results illustrated in Figure 2, show c-Fos activity in the DG following test, which took place two weeks after CFC training (Fig. 1, experimental timeline). As expected, only a small fraction of the general granule cell population expressed c-Fos in control animals (Fig. 2*A*). A similar result was obtained for the CA1 field of the hippocampus (Fig. 2-1, Extended data).

10.1523/ENEURO.0237-17.2017.1 Figure 2-1 Activity-dependent regulation of c-Fos in the CA1 area of the hippocampus. Number (mean ± SEM) of c-Fos-labeled cells per CA1. Tested rats had significantly more cFos+ cells per CA1 compared to controls. Two-way ANOVA shows an effect o*f* testing (*F*_(3,79)_ = 10.838, *p = 0.001). There was no effect of running (*F*_(1,79)_ = 1.131, *p* = 0.291) and no interaction (*F*_(3,79)_ = 2.151, *p* = 0.101). Download Figure 2-1, TIF file.

A two-way ANOVA comparing c-Fos activation within the DG in early runners versus non-runners shows a testing-induced increase in c-Fos (*F* = 15.9, *p* < 0.001) that was equal in runners and non-runners (*F*_(1,79)_ = 0.297, *p* = 0.588) with no interactions among the variables (*F*_(1,79)_ = 0.063, *p* = 0.979; Fig. 2*B*). On subsequent examination of different contexts, the results showed that this testing-induced increase in the number of c-Fos+ cells generalized to all contexts, as shown by similar levels of activation in all tested subgroups in both running and non-running conditions (Fig. 2*C*). These results are a robust indication that DG and the hippocampus as a whole participates in the CFC task and that early running does not influence this activity.

Additional estimates of granule cell volumes revealed no significant differences among the groups (two-way ANOVA using running (*p* = 0.116) and testing (*p* = 0.883) as variables; data not shown). Thus, the test-induced increase in the hippocampal c-Fos activity is a true measure of the increased activity among the pre-existing neurons and not an effect on hippocampal growth.

### c-Fos activity is related to adult-born neurons and enhanced by running

To determine if the c-Fos activity was related to adult-born granule neurons rather than to the pre-established general population, the densities of double-labeled cells (c-Fos/CldU) were measured. The animals were injected with CldU three weeks before training thus labeling a cohort of five-week-old neurons at the time of testing. This age was chosen to ensure a full maturity and functionality of the neurons in terms of IEG expression (Snyder et al., 2009).

**Figure 3.**
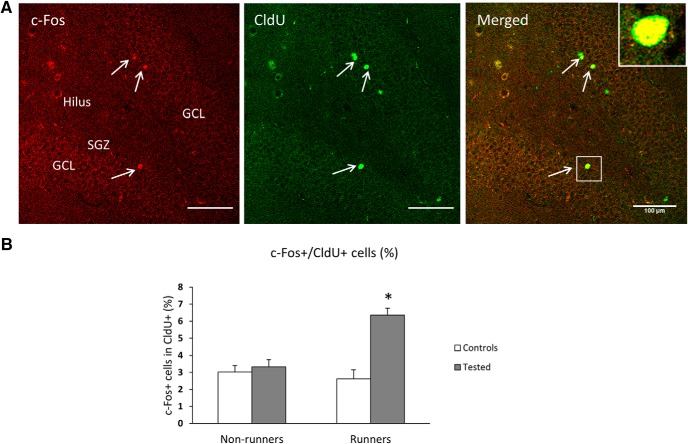
Neuronal activity in adult-born neurons in runners and non-runners. ***A***, Confocal microscopic images showing c-Fos- and CldU-labeled cells within the subgranular zone (SGZ) of the GCL. White arrows indicate dual-labeled c-Fos+/CldU+ cells in the GCL. Boxed area is enlarged in the inset showing the double-labeled cell. Scale bars, 100 µm. ***B***, Graph showing % expression of c-Fos+ cells in CldU+ cells within DG in control and tested groups. There is a significant effect of running in memory tested group (*p < 0.05, *n* = 10 rats/control group; *n* = 30 rats/memory-tested group). No difference in cell numbers between control and tested rats in non-runners.

The estimates demonstrate that the proportion of active cells is ∼3% in cage controls, and up to 7% in tested runners (Fig. 3*B*). In terms of absolute numbers, the c-Fos+/CldU+ cells (non-runner controls, mean = 20.4; SD = 3.5/DG) were less numerous than the c-Fos+ cells in the general DG population (compare to Fig. 2). However, the percentage activation numbers show that, not only are the adult-born neurons more active than a general population but also their activity is significantly enhanced in early runners as compared to non-runners in tested animals (two-way ANOVA for running (*F*_(1,79)_ = 5.837, *p* = 0.018), testing (*F*_(1,79)_ = 13.946, *p* = 0.001) and (*F*_(1,79)_ = 10.115, *p* = 0.002) interactions; Fig. 3*B*).

**Figure 4.**
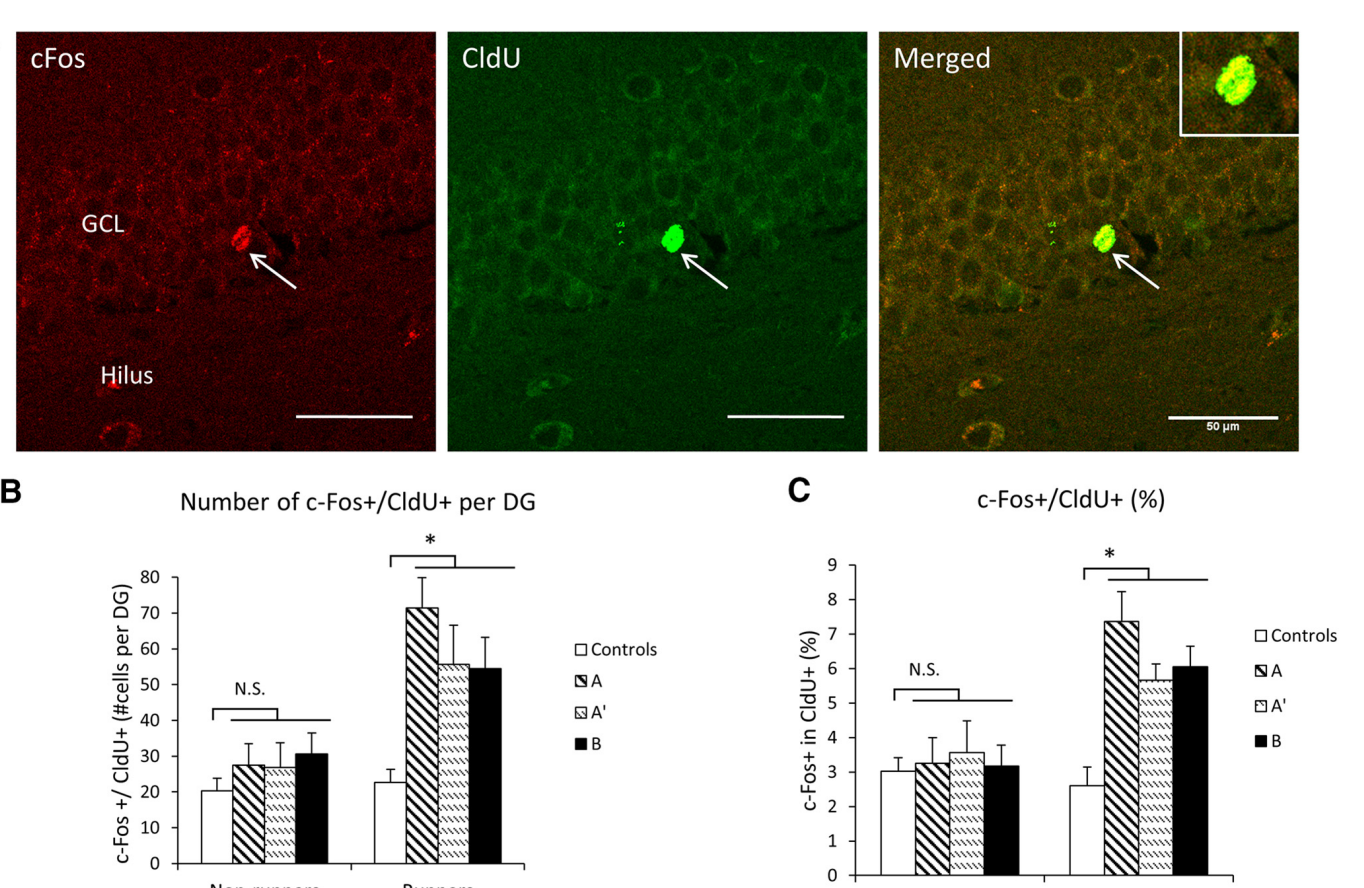
c-Fos activity in adult-born neurons is enhanced by running. ***A***, Representative images of c-Fos+/CldU+-labeled cells within the GCL. White arrow indicates dual-labeled cells. The inset shows a high magnification view of the c-Fos+/CldU+-labeled cell. Scale bars, 50 µm. ***B***, Absolute numbers of double-labeled c-Fos+/CldU+ cells. Comparison o*f* tested groups (controls, context A, A’, and B) within runners and non-runners. All three tested groups have significantly more cell numbers compared to controls (*p < 0.05, *n* = 10 rats/control group; *n* = 30 rats/memory tested group). The numbers of CldU+ cells did not differ in any of the groups (Fig. 4-1, Extended data). ***C***, Graph showing % expression of c-Fos in CldU cells. Controls, non-tested cage controls; A, tested in the familiar environment; A’, tested in the similar environment; B, tested in the novel environment. The percentage of c-Fos expression in CldU cells was greater in all tested groups compared to controls in runners (*p < 0.05, *n* = 10 rats/group).

Next, c-Fos activity was examined in relation to specific tasks (A, A’, or B) administered to animals at the time o*f* testing. Context A representing the environment in which the rats were trained, A’ representing a similar, and B a completely novel environment (Fig. 4).

10.1523/ENEURO.0237-17.2017.2 Figure 4-1 Cell survival in the DG. Graph showing number (mean ± SEM) of CldU-labeled cells per DG. No difference in the number of CldU+ cells was detected between groups. Two-way ANOVA shows no effect of running (*F*_(1,79)_ = 1.749, *p* = 0.19), testing (*F*_(1,79)_ = 0.56, *p* = 0.453) and no interactions. Download Figure 4-1, TIF file.

Two-way ANOVA performed on the absolute numbers of the double-labeled cells shows significant effects of running (*F*_(1,79)_ = 23.57, *p* < 0.001), testing (*F*_(3,79)_ = 5.582, *p* = 0.002), and significant interaction (*F*_(3,79)_ = 2.872, *p* = 0.042; Fig. 4*B*). In particular, the three tested groups were higher in comparison to controls (pairwise comparison at *p* < 0.05). Also, the cell numbers in tested runners were higher in comparison to those in tested non-runners. There were no differences among any of the non-running groups. Thus, the early-age running specifically enhances the activity of adult-born five-week-old neurons when rats are tested in the same, familiar or novel environment. Importantly, the percentage c-Fos expression is elevated in parallel to the absolute cells numbers. The increase shown in Figure 4 is not due to enhanced cell survival in tested runners since the numbers of CldU+ cells did not differ in any of the groups (Fig. 4-1, Extended data).

### Memory selectivity is enhanced by running

**Figure 5.**
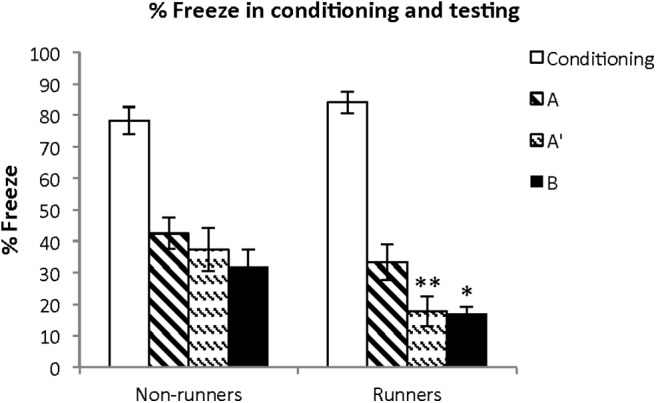
Results of CFC training and testing. Early running did not affect acquisition of the CF response during training. There were no differences among the group A (familiar environment) tested animals. The group A’ (similar environment) showed significantly (**p = 0.01) less freezing in runners. The group B (different environment) showed significantly (*p < 0.05) less freezing in runners.

The animals were administered CF conditioning in context A two weeks before perfusion (Fig. 1, experimental timeline). The performance of all groups following CF conditioning and testing are presented in terms of percentage time the animals spent freezing (Fig. 5). Two-way ANOVA of freezing times and running showed a significant effect of running (*F*_(1,54)_ = 11.672, *p* < 0.001) and testing (*F*_(2,54)_ = 3.692, *p* = 0.031). The interactions between running and testing could not be adequately examined due to a low statistical power (0.05); however, a pairwise comparison with the Bonferroni *t* test revealed a significant difference between runners and non-runners within context A’ (*p* = 0.01) and B (*p* = 0.049) but not within A (*p* = 0.219).

These effects were specific for memory testing since the conditioning (training) phase showed no difference in learning. The percentage freezing scores in runners (84.2, SE = 3.5) and non-runners (78, SE = 4.3) were not different (*t* = 1.027, 58 df, *p* = 0.31). In summary, these behavioral results show no effect of running on memory of the familiar context (A) but reduced freezing in the similar (A’) and completely different (B) contexts. The results parallel the c-Fos activation in adult-born granule DG neurons showing selective activation in runners but not in non-runners.

## Discussion

The most striking result of this study is the effect of early-age running on memory-related neuronal activity (Figs. 3, 4). This is the first evidence of such a long-term effect and a confirmation of an animal model of cognitive reserve. The model was based on numerous human studies which used a variety of forms of enrichment to induce the reserve phenomenon reviewed in (Merkley and Wojtowicz, 2016). Running is known to reliably induce increases in adult neurogenesis and enhance cognitive function in animals and in humans (Déry et al., 2013; Hutton et al., 2015). The six-week period of running was based on a previous study and is sufficient to produce a maximal effect since running reached a maximum at that period (see Materials and Methods). A relatively high 6-7% of c-Fos in adult-born neurons, as shown in Figure 3, is far greater than the sparse activation of <1% seen in the developmentally born neurons in agreement with previous estimates (Chawla et al., 2005; Ramirez-Amaya et al., 2006) and our data in Figure 2.

In the present study, rats in the running condition, that were found to exhibit enhanced neurogenesis, also froze less than non-runners when tested in contexts A’ and B indicating better discriminability of the contextual environments and less generalization of the fear response. There is considerable evidence that the ability to discriminate between overlapping stimulus elements (pattern separation), is associated with hippocampal function (Leutgeb et al., 2007) and, in particular, adult neurogenesis in DG (Clelland et al., 2009; Abrous and Wojtowicz, 2016; McAvoy et al., 2016). The present results extend previous reports by showing that adult neurogenesis can be enhanced by early experience and, as a mechanism underlying pattern separation, can be mediated through a build-up of cognitive reserve. The enduring effects of early running may have occurred at the level of stem cells and/or the neurogenic milieu, presumably in the subgranular zone of the DG. Hippocampal afferents are known to influence the neurogenic zone by stimulating distinct phases of cellular growth and development (Wojtowicz and Tan, 2016) and epigenetic influences on progenitors that could mediate such changes have been described (Ma et al., 2009; Yao et al., 2016).

Runners tested in context A also exhibited more activity in adult-born cells than non-runners, but the groups did not differ in freezing time. This outcome was not necessarily expected but is understandable when considering the dynamic changes that occur in CF memory over time (Winocur et al., 2010). Several studies have shown that CF memories, when initially encoded in the hippocampus, are highly context specific and do not generalize to other environments. After a few days, these memories transform into less specific schematic memories that can be evoked by a sample of the original contextual cues or even by new contexts that bear only a slight similarity to the original (Wiltgen and Silva, 2007; Winocur et al., 2007). In the normal animal, the context-specific and non-specific versions can coexist and the type of memory that is expressed depends on several factors (Winocur et al., 2013). It is likely that, in the present study, the runners, with elevated neurogenesis activity and enhanced hippocampal function, were expressing hippocampus-dependent, context-specific memory of the fear memory, whereas the non-runners were retrieving the more general, schematic version that is believed to be represented in a network of cortical regions.

The influence of early life experiences on learning and memory in later life is a topic needing more study (Stern, 2012). Numerous human studies point to beneficial effects of educational and/or physical experiences during youth on symptoms of cognitive decline in old age. The idea was publicized by the “Nun study” which described several examples of such life-long effects (Iacono et al., 2009). As well, in line with the cognitive reserve hypothesis, other survey-type studies have emphasized the impact of early age experiences on cognitive performance in late adulthood (Scarmeas and Stern, 2003; Petrosini, 2009). Qualitatively, cognitive reserve can be viewed as a buffer or reservoir of plasticity acquired early in life, that is used later in response to normal age-related decay or pathologic damage. As such, cognitive reserve may constitute an important part of the compensatory mechanisms frequently observed in the compromised brain (Wang et al., 2005).

There is considerable evidence that early physical and cognitive activity can delay the onset of pathologic changes that accelerate the process of cognitive decline in old age (Valenzuela and Sachdev, 2006; Stern, 2012). In animal models of Alzheimer’s disease (AD) changes in the adult neurogenesis often precede appearance of pathologic markers and cognitive symptoms of AD (Hamilton et al., 2010; Krezymon et al., 2013). Thus, reduced levels of adult neurogenesis may be a measure of early symptoms and a predictor of subsequent disease. If so, it follows that early lifestyle related interventions (e.g., social stimulation, physical exercise) that help to prevent such effects through the build-up of a neurogenic reserve (Merkley and Wojtowicz, 2016), may provide a measure of protection against early cognitive impairment.

## Synthesis

Reviewing Editor: Liisa Galea, University of British Columbia Decisions are customarily a result of the Reviewing Editor and the peer reviewers coming together and discussing their recommendations until a consensus is reached. When revisions are invited, a fact-based synthesis statement explaining their decision and outlining what is needed to prepare a revision will be listed below.

This is a well written study examining the effect of postweaning running throughout puberty in male rats on contextual fear conditioning, neurogenesis and activation of 5 week old neurons in adult rats. I have only minor corrections for the authors.

1) Please ensure in the title and in the Methods section that it is clear only male rats were tested.

2) line 50: ‘This study...’ it is not clear which study authors are referring to there.

3) line 52 - I would change enrichment to ‘voluntary running’

4) line 108 - cohorts are mentioned but not described.

5) Please give some indication of the variability in running between animals.

6) lines 200-204 - Please give the factor names for the 2-way ANOVA - Running and testing

7) line 249 - after non-runners - don't you need to say only the ‘tested’ animals?

8) line 259 - interactions should not be plural as only one

9) line 275 - I think you mean p>0.05 if not significant

10) line 276-282: Multiple t-tests should not be used - in the case of a non-significant interaction a priori tests can be conducted and care needs to be taken to avoid type 1 errors ( need a correction for multiple t-tests).

Might consider adding graphs that have not just means but individual data points.
