# Supplementary figures and images for: Early-Age Running Enhances Activity of Adult-Born Dentate Granule Neurons Following Learning in Rats
Source: eNeuro. 2017 Aug 16;4(4):ENEURO.0237-17.2017. doi: 10.1523/ENEURO.0237-17.2017 (PMC5560743; doi:10.1523/ENEURO.0237-17.2017)

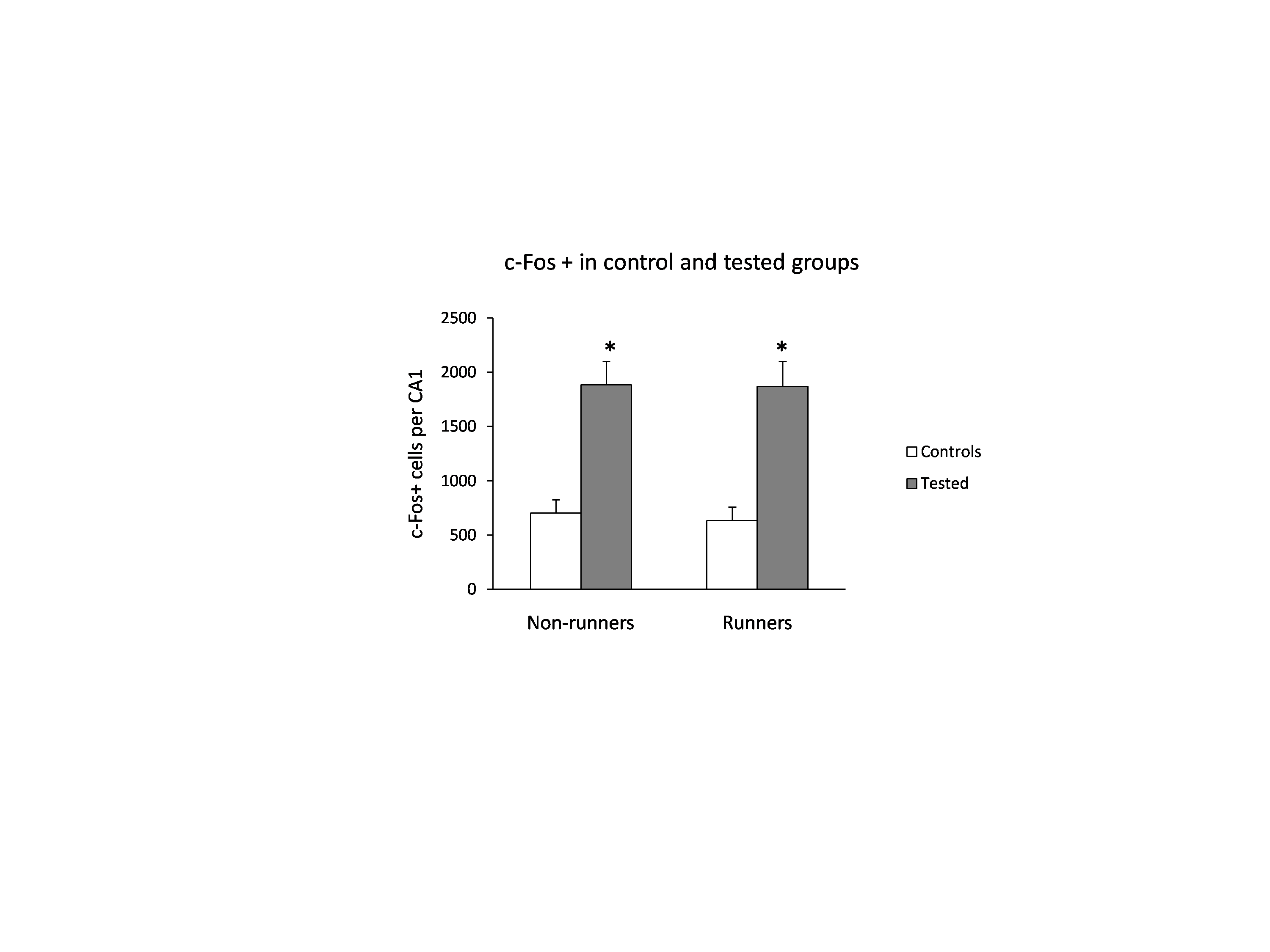

Supplement: Figure 2-1 [file enu004172375so6.tif]

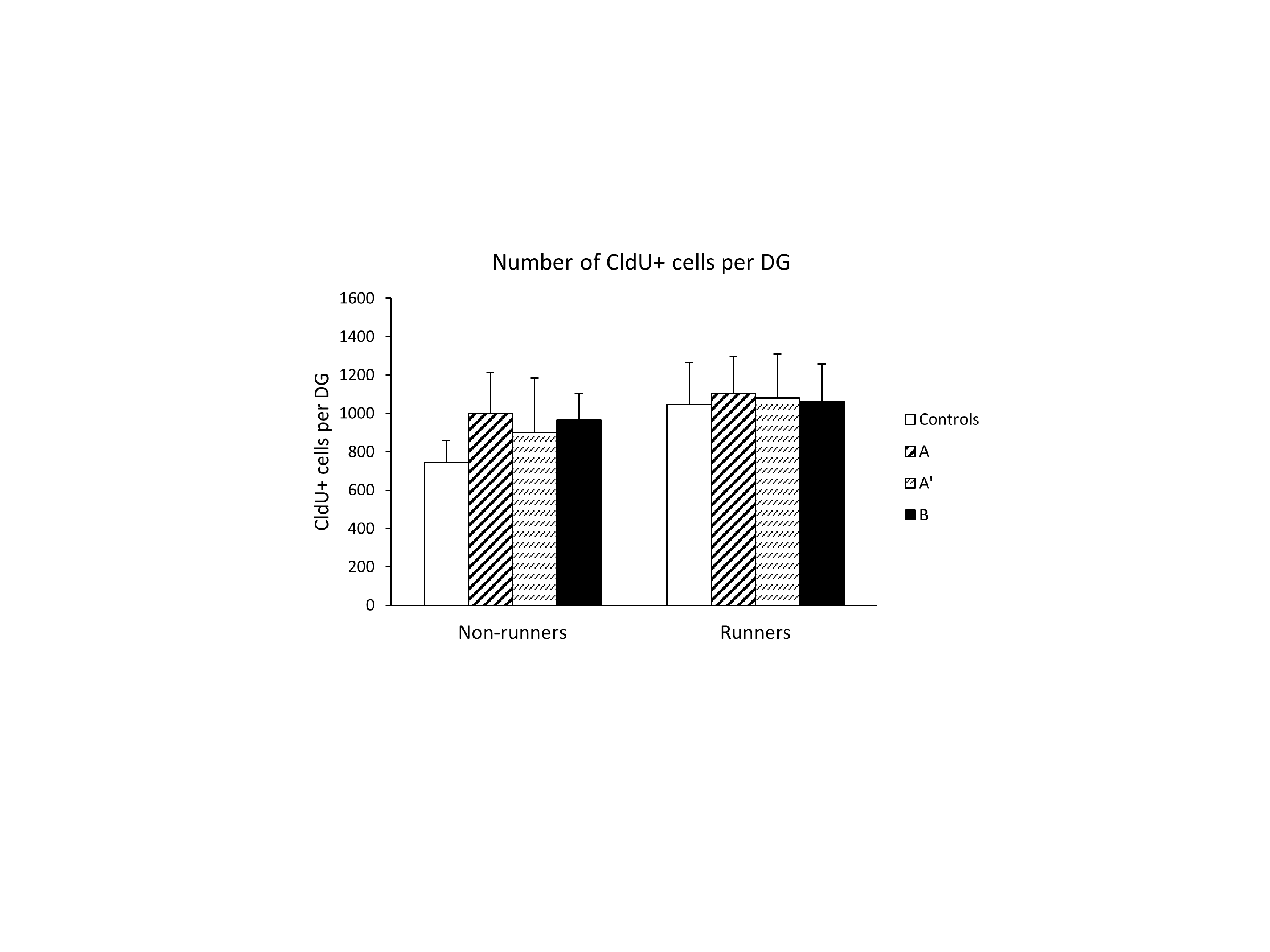

Supplement: Figure 4-1 [file enu004172375so7.tif]
